# Toward a Neuroscientific Understanding of Play: A Dimensional Coding Framework for Analyzing Infant–Adult Play Patterns

**DOI:** 10.3389/fpsyg.2018.00273

**Published:** 2018-03-21

**Authors:** Dave Neale, Kaili Clackson, Stanimira Georgieva, Hatice Dedetas, Melissa Scarpate, Sam Wass, Victoria Leong

**Affiliations:** ^1^Department of Psychology, University of Cambridge, Cambridge, United Kingdom; ^2^School of Education, University of Delaware, Newark, DE, United States; ^3^Division of Psychology, University of East London, London, United Kingdom; ^4^Division of Psychology, Nanyang Technological University, Singapore, Singapore

**Keywords:** play, mother–infant interaction, neuroscience, coding, social interactions

## Abstract

Play during early life is a ubiquitous activity, and an individual’s propensity for play is positively related to cognitive development and emotional well-being. Play behavior (which may be solitary or shared with a social partner) is diverse and multi-faceted. A challenge for current research is to converge on a common definition and measurement system for play – whether examined at a behavioral, cognitive or neurological level. Combining these different approaches in a multimodal analysis could yield significant advances in understanding the neurocognitive mechanisms of play, and provide the basis for developing biologically grounded play models. However, there is currently no integrated framework for conducting a multimodal analysis of play that spans brain, cognition and behavior. The proposed coding framework uses grounded and observable behaviors along three dimensions (sensorimotor, cognitive and socio-emotional), to compute inferences about playful behavior in a social context, and related social interactional states. Here, we illustrate the sensitivity and utility of the proposed coding framework using two contrasting dyadic corpora (*N* = 5) of mother-infant object-oriented interactions during experimental conditions that were either non-conducive (Condition 1) or conducive (Condition 2) to the emergence of playful behavior. We find that the framework accurately identifies the modal form of social interaction as being either non-playful (Condition 1) or playful (Condition 2), and further provides useful insights about differences in the quality of social interaction and temporal synchronicity within the dyad. It is intended that this fine-grained coding of play behavior will be easily assimilated with, and inform, future analysis of neural data that is also collected during adult–infant play. In conclusion, here, we present a novel framework for analyzing the continuous time-evolution of adult–infant play patterns, underpinned by biologically informed state coding along sensorimotor, cognitive and socio-emotional dimensions. We expect that the proposed framework will have wide utility amongst researchers wishing to employ an integrated, multimodal approach to the study of play, and lead toward a greater understanding of the neuroscientific basis of play. It may also yield insights into a new biologically grounded taxonomy of play interactions.

## Introduction

### Challenges to a Neuroscientific Understanding of Play

Play during early life is a ubiquitous activity. Engaging in play is positively associated with the development of social skills, cognitive skills, language and emotional well-being (Lyytinen et al., 1999; Pellegrini et al., 2002; St George et al., 2016; Thibodeau et al., 2016; Fung and Cheng, 2017). Current conceptualizations of play in the behavioral sciences view it in broad terms as behavior that is voluntary, engaging, non-functional, and associated with the expression of positive affect (Burghardt, 2005; Lillard et al., 2013; Miller, 2017). Play can also be categorized based on the focus of play, i.e., what is the individual playing with? For example, *physical play* is play with one’s own body and other people, for example, climbing, sliding, chasing (Power, 1999; Pellegrini et al., 2002; St George et al., 2016); *sociodramatic* or *pretend play* is play with a make-believe world, and the focus of play is more than a concrete observable entity (Lillard et al., 2013); *games with rules* involve playing with a set of rules that participants agree to abide by to partake in the play experience, for example, board games or playground games such as tag (Hassinger-Das et al., 2017); and *object play* involves playing with physical objects (Power, 1999; Pellegrini and Gustafson, 2005). Object play can be further sub-divided, depending on the activity conducted with the object. For example, objects may be used in relational play, where multiple objects are combined or joined together, and object-pretense, where the object is used to represent something else (Belsky and Most, 1981).

These diverse categorizations and definitions show that play in humans is diverse, multi-faceted, and defined by a set of broad terms encompassing motivational, cognitive, social and emotional aspects of behavior and psychology. Consequently, a challenge for current research is to converge on a common definition and measurement system for play – whether examined at a behavioral, cognitive or neurological level. This is a timely challenge to address as advanced brain imaging techniques now permit the concurrent capture of neural activity from adult–infant dyads during naturalistic social interactions, such as joint play (Wass and Leong, 2016; Leong et al., 2017). Central to this challenge is the fact that it is difficult to exert the level of experimental control and temporal precision required for investigation at the neurological level, while also retaining the freeform, diverse quality which many consider to be a defining feature of play.

### Development of Human Play Behavior

Play behavior changes substantially across the life-span (Power, 1999). These changes in play behavior occur per one’s developmental level (e.g., progression from solitary play to cooperative play) and interactions with others in an effort to achieve developmental goals. During infancy, mothers engage in one-on-one play with their baby to model and promote skills necessary for their child’s development; such as communication and language skills, increase their cognitive capacities, foster autonomous development, and other important skills that are required for social interaction and well-being (e.g., Valentino et al., 2011; Bernier et al., 2016). For example, Mermelshtine and Barnes (2016) found that the mother’s responsiveness to their infant during play at 10 months of age positively predicted higher cognitive capacities and skills (e.g., problem solving, knowledge and memory) at 18 months. This effect remained after accounting for maternal education, home adversity and infant advanced object play. While dyadic interaction e.g., mother-infant – is present throughout the first year of life, it is not until around the end of the first year that infant’s ability to engage in triadic interaction (i.e., mother-infant-object) becomes consolidated (Bakeman and Adamson, 1984; de Barbaro et al., 2013a). This progression to triadic interaction, focussed around an object, is considered important for many aspects of psychological development, including symbolic awareness and language (Tomasello, 1999; De Schuymer et al., 2011). As Rodríguez (2009) points out, objects are symbols of their uses within a culture (a cup, for example, can represent drinking), and an understanding of these object-use relations represents the early acquisition of cultural norms and adoption of a fundamental symbolic system. Adults and infants communicate *about* objects and *with* objects. Furthermore, there is substantial crossover between the literatures on object play and object exploration in infancy, and exploration is viewed as a fundamental part of early childhood play (Belsky and Most, 1981). The evidence suggests that object exploration in infancy plays a role in the development of problem-solving and attention (Caruso, 1993; Poon et al., 2012; Clearfield et al., 2014) and individual differences are observed between children from different socioeconomic backgrounds (Clearfield et al., 2014). Consequently, focusing our model around a physical object was deemed the best approach for studying behavioral and neural activity during parent–infant play.

In addition to using object play in the current approach, the context of mother-infant play is equally important for the current study. The importance of mother-infant interactions on early development is well documented (e.g., Belsky and de Haan, 2011; Bernier et al., 2016; Mermelshtine and Barnes, 2016). However, the interactions measured are often related to parenting processes (e.g., parental support/affect, sensitivity, communication, responsiveness) that occur in a play context and do not include quantitative coding of the actual play interactions. While these studies do provide insights into how early development is influenced by maternal parenting processes, they cannot explain the specific role of mother-infant play. Therefore, it is an important next step to examine if and how mother–infant play affects early development, particularly neural development. In order to achieve this goal, a play coding scheme that is compatible in time-resolution to that of brain imaging measures should be developed.

### Insights Into the Neuroscience of Play From Animal Models

Due to the challenges of experimental control (e.g., standardization of participants’ behavior and environment), neuroscience studies on play have primarily focused on animal models (in particular rats) and rough-and-tumble social play behavior (see reviews by Pellis and Pellis, 2009; Cooke and Shukla, 2011; Siviy and Panksepp, 2011; Vanderschuren et al., 2016). Rodent models have proven to be particularly useful because rats show predictable and stereotypical forms of play-related behavior (e.g., one animal ‘pins’ the other on its back, emission of ultrasonic vocalizations, etc.) which are readily quantifiable and amenable to experimental and pharmacological manipulation. Consequently, a relatively rich literature now exists on the neuroanatomical and neurochemical substrates of rough-and-tumble play behavior in rats, using (invasive) methods such as brain lesioning, intracranial administration of neuroactive compounds, and gene expression assays. Namely, the key neural circuits that are now known to work in concert to support rats’ play fighting behavior are: (1) a *cortical executive circuit* (particularly the prefrontal cortex (PFC) and orbitofrontal cortex (OFC)) which mediates the developmental fine-tuning and complexity of play, such as the ability to coordinate with or modify movements in response to the social status of a play partner (Moore, 1985; Pellis et al., 1999; Pellis et al., 2006; Bell et al., 2009; Siviy and Panksepp, 2011); (2) a *subcortical limbic circuit* (amygdala, hypothalamus and striatum) which moderates the motivation for, and affective response to play (Meaney et al., 1981; Wolterink et al., 2001; Daenen et al., 2002; Burgdorf et al., 2007), potentially via dopaminergic and opioid pathways (Vanderschuren et al., 2016); and (3) *somatosensory circuits* (somatosensory cortex, thalamus, cerebellum) which control motor play patterns and performance (Siviy and Panksepp, 1985, 1987a,b; Panksepp et al., 1994; Byers and Walker, 1995).

Animal studies have further shown that play induces neural plasticity in brain areas involved in sensorimotor processing (e.g., parietal cortex, colliculi and striatum, Gordon et al., 2002), and also in the medial prefrontal cortex (mPFC, Cheng et al., 2008), an area which sends strong modulatory inputs to limbic circuits that control social behavior. In humans, the mPFC inhibits aggression and monitors approach/avoidance behavior (Bufkin and Luttrell, 2005; Hall et al., 2010). Therefore, increased plasticity in the mPFC following play could indicate that play helps to improve control of social behavior networks. Rough-and-tumble play in rats also seems to promote brain development by increasing the expression of brain-derived neurotrophic factor (BDNF) in the amygdala and prefrontal cortex (Gordon et al., 2003), and that of insulin-like growth factor 1 (IGF-1) in the frontal and posterior cortices (Burgdorf et al., 2010). Accordingly, it has been suggested that play-induced neural plasticity could support the emergence of adult-like behaviors (Cooke and Shukla, 2011). Although caution must be applied in extrapolating findings from animal work to humans, the current data do suggest that across species, play may be a fundamental neurobehavioral process that is underpinned by (and produces changes in) major cortical and subcortical neural circuits that support cognition, emotion and sensorimotor function. However, the neuroscientific methods that have successfully been used with animal models are too invasive to be performed on human subjects. Further, even when ostensibly comparing “motor-based” play, human play behavior is far more complex and less stereotypical than animal play-fighting behavior, as described above. Consequently, neuroscience research into play in humans tends to either assess neurological change using a pre-test, post-test design (Newman et al., 2016), or to study neural activity while the participant observes, but does not engage in, play behavior (Smith et al., 2013). Going beyond these empirical constraints to identify the neural mechanisms that underlie ongoing, complex play behavior in humans presents a considerable challenge. To address this challenge, non-invasive human neuroimaging (e.g., EEG, fMRI) and psychological behavioral coding approaches could be combined into a multimodal analysis that may yield advances in understanding the human neurocognitive mechanisms of play. However, there is currently no methodological framework that is suitable for conducting a multimodal analysis of play that spans brain, cognition and behavior.

### Limitations of Current Measures of Play for Neural Analyses

In order to combine neural and behavioral analyses of play, the behavior of interest must be identified with precise temporal resolution. In addition, the behavioral coding should be able to identify change between various play and non-play states, to facilitate the intra- and inter-individual analysis of corresponding changes in neural activity. However, existing play coding schemes are predominantly based around global ratings, checklists, or frequency counts of play behaviors, and so do not capture temporal information about *when* specific play behaviors occur, or information about non-play behavior. Examples of global rating schemes include Poon et al. (2012) who rated parent-infant play sessions on a scale of 1 – 5 for joint attention, imitation and object play, and St George et al. (2016) who gave each parent a global score on 10 different dimensions, including sensitivity (how responsive the parent was to the child’s signals), positive regard (demonstrations of love and affection), and stimulation of cognitive development (teaching). Check-list approaches include the Symbolic Play Test (Lowe and Costello, 1976), which captures behaviors which children display when playing with a specific set of toys, such as ‘feeds doll’ and ‘moves truck or trailer about.’ A similar checklist approach is found in many bespoke measures of play, such as that used by Pellegrini (1992), where children were observed in the playground and the behaviors displayed were recorded, including *peer interaction* and *object play*. In an analysis of infant play, Belsky and Most (1981) applied a checklist approach to time-sampled data, by using a checklist to record the ‘most competent’ level of play observed in each 10-s period. The authors acknowledge that this approach obscures information about the frequency of play behaviors, as any ‘lower level’ play behaviors occurring in the same 10-s period cannot captured by the coding scheme. But information about ‘high level’ behaviors is also obscured, including their precise timing and frequency within each 10-s period. From a neuroscience perspective, knowing that one type of play occurred at some point within a 10-s window does not provide sufficient temporal precision for event-locked analyses to be conducted.

A few studies have captured more precise temporal information in the context of mother/parent–infant play (e.g., Courage et al., 2010; James et al., 2012; Zuccarini et al., 2017). However, no play-specific coding schemes that we are aware of measure play behavior between the parent and infant at a time resolution that is fine-grained enough (i.e., 10s of milliseconds) to be compatible with neural (e.g., EEG) analyses. Furthermore, these existing schemes are not designed to capture and analyse the temporal evolution of a range of behavioral states as a fluid continuum. For example, Zuccarini et al. (2017) coded ‘motor object exploration’ in infant play, where the infant explored an object with their hands or mouth, and Koterba et al. (2014) coded infant looking and mouthing during play with a rattle. While such schemes reflect our emphasis on the continuous fine-grained coding of play behavior, they do so by coding one or two specific actions and then analyzing how the duration or frequency of those actions vary between infants. Our coding scheme, by contrast, is designed to track the continuously evolving behavior of participants as they move through play, teaching/learning, joint attention, and other such states, and facilitate analysis within, as well as between, participants. A multimodal coding system for mother–infant play, suitable for analyzing co-occurring patterns in real-time behavioral and neurological data, requires the flexibility to capture a wide variety of behavioral states, combined with a very high degree of temporal precision, and such a combination does not exist in established play coding schemes.

Second (as indicated by animal studies), play is a highly complex social interactive activity that activates a combination of sensorimotor, cognitive and socio-emotional neural circuits - each of which may support separable dimensions of behavior. Importantly, no single behavioral dimension by itself is sufficient to define play, since behavior in each dimension can occur in both playful and non-playful situations. Rather, it is the co-occurrence of activity along multiple dimensions that defines a playful episode. Here, we contribute to the formation of a neuroscientific understanding of play by presenting a model and methodological framework that captures behavior at a high temporal resolution and as a continuously evolving multi-dimensional state, rather than as a set of discrete actions or as a global summary of type or quality. In this way, behavioral coding is well matched to the high temporal resolution of EEG data, maximizing the acuity with which brain-behavior correlates can be explored.

### Overview and Considerations of the Play Coding Methodological Framework

As described in the previous section, current neuroscientific research suggests that play behavior is underpinned by three major neural circuits that control motivation and affect (i.e., limbic structures), motor performance (i.e., somatosensory structures), and higher-order executive function (i.e., frontal cortical structures) respectively. Following from this, the proposed coding framework captures object-oriented play behavior along three corresponding dimensions: socioemotional (SE), sensorimotor (SM), and cognitive (C). Infants’ or adults’ behavior is coded according to the presence or absence [1/0] of play-congruent activity in each dimension. The intention of the coding scheme is to reliably capture common forms of playful behavior whilst retaining clarity of coding for each dimension (grounding the scheme in clear, observable, behaviors). With this in mind, play-congruent activity was defined for each dimension as follows:

Play-congruent activity in the SE dimension occurs when there is a display of positive or neutral affect, consistent with the idea that play leads to an internal sense of reward (Burghardt, 2005; Miller, 2017). Play-congruent activity in the SM dimension occurs when the partner (mother or infant) is voluntarily manipulating and/or touching the object in an exploratory manner. This criterion reflects the central place of self-directed, voluntary behavior in definitions of play (Burghardt, 2005; Lillard et al., 2013; Miller, 2017; Sawyer, 2017). Finally, the C dimension captures the presence of attentional engagement, as well as the level of complexity of this cognitive engagement. Therefore, our analysis is intended to explore ‘minds-on’ play, rather than ‘minds-off’ play. By ‘minds-on play,’ we mean play where cognition and attention are engaged through, for example, observation of object/partner behavior, exploration of the object, or communication. ‘Minds-off play,’ by contrast, refers to play behavior where cognition and attention disengage with the play object and partner, and the play goal is more sensory in nature, for example, chewing a toy or hitting it on the table while looking elsewhere. According to our framework, a *play-congruent* state is one in which the infant (or adult) concurrently exhibits play-congruent activity across all 3 dimensions (i.e., [1 1 1]).

An important feature of this framework is that it does not assume any one definition of play. Instead, we have grounded the framework in specific observable behaviors that are considered important factors across different conceptualizations of play – namely, the display of affect and voluntary physical and cognitive engagement with the object of play (Lillard et al., 2013; Miller, 2017). By analyzing the co-occurrence patterns of these basic behaviors, our framework can be used to assess similarities and potential groupings of different play-related social states (and, eventually, their neural substrates), which may in future lead to a definition of play behavior that is grounded in neuroscience. Another strength of the proposed framework is that it reduces the burden of subjective judgment about whether or not playful activity is occurring. Rather, objective and observable behaviors are coded (e.g., touching a toy, looking at a toy, smiling, etc), and the presence or absence of play (and other related social states) is inferred from temporally co-occurring patterns of behavior. Grounding coding in specific observable behaviors tends to result in higher levels of inter-rater agreement (Bakeman and Gottman, 1997).

### Aims and Predictions

The goal of the current study is to develop a new methodological framework for coding infants’ and adults’ playful behavior that would be compatible, in future, with EEG analysis. As mentioned previously, current research on mother-infant interactions and infant development often measure the parenting processes that occurs within a play context rather than the play itself, and current mother-infant play coding schemes typically lack the temporal precision to be integrated with neural measures. Therefore, we illustrate the application of our proposed dimensional play coding framework using examples from two contrasting dyadic corpora of mother–infant object-oriented interactions during experimental conditions that were either non-conducive (Condition 1) or conducive (Condition 2) to eliciting playful behavior. In Condition 1, playful behavior was discouraged by asking mothers to focus on teaching infants about the social value (desirable or non-desirable) of the objects. In Condition 2, playful behavior was encouraged by asking mothers to use the objects in spontaneous, fun and natural interactions with their child. These corpora comprise both behavioral and electroencephalography (EEG) measurements that were collected concurrently from mothers and their infants. However, for this study, we focus on behavioral analyses. It is intended that the coding of play behavior under the proposed methodological framework will be easily assimilated with, and inform, future analysis of neural data that was also collected during adult-infant play. We have two specific sets of predictions regarding the behavioral differences between conditions that should emerge following application of the coding framework:

(1) In Condition 2 (conducive), infants’ modal state will be [1 1 1] (i.e., play-congruent along all three dimensions), but in Condition 1 (non-conducive), [1 1 1] will *not* be the modal state;
(2) In Condition 2 relative to Condition 1, infants will show:
  (a) Decreased negative affect
  (b) Increased sensorimotor engagement
  (c) Equivalent cognitive (attentional) engagement

The first prediction pertains to the *sensitivity* of the coding framework in detecting play-related behavior. Simply put, if mothers were instructed to play with their infants, then (although coders do not make direct judgments about whether participants were playing or not) we expect the coding framework to reveal that a play-congruent state was indeed the most frequent social state that infants displayed. The second set of predictions pertains to the *utility* of the framework in identifying differences in the quality of social interaction and temporal synchronicity with the dyad.

## Materials and Methods

### Participants

Five mother–infant dyads participated in the study (3M, 2F infants). Infants were aged 326.6 days (10.7 months) on average [range = 292–377 days (9.6–12.4 m), SD = 31.5 days (1.0 m)]. All mothers reported no neurological problems and normal hearing and vision for themselves and their infants. Although this sample size appears small, note that the aim of the present study was to assess how well the coding scheme captures intra-dyadic variation (variation in dyadic states over time and between conditions) rather than inter-dyadic variation (variation between dyads). Further, each dyad participated in two different conditions and generated over 20 min of data, which is substantial for infancy studies.

### Materials

For Condition 1 (non-conducive to play), 4 pairs of ambiguous novel objects were used. Within each pair, objects were matched to be globally similar in size and texture, but different in color. Ambiguous novel objects were chosen to ensure that infants would not have their own previous (playful) experience with these objects, and would rely on their mothers’ instruction to guide their interactions with the objects.

For Condition 2 (conducive to play), a set of 8 different small toys was used. These were appropriate for the infants’ age and included toys of differing shapes, textures and colors to encourage infants’ interest in playing with them.

### Tasks

Each mother-infant dyad took part in experimental Conditions 1 and 2 in a counterbalanced order. In each condition, mothers and infants interacted with objects together, with the major difference being whether the nature of social interaction between mother and infant was conducive to eliciting playful behavior (as determined by the task instructions provided to the mother). In both tasks, the infant sat in a high chair, with the adult facing him/her across a table. The distance between the infant and adult was the same in each task, and each task lasted approximately 10 min.

#### Condition 1 (Not Play-Conducive)

**FIGURE 1:**
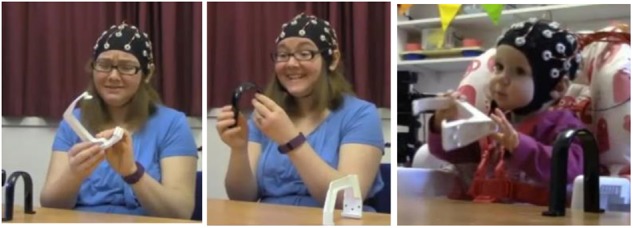
Illustration of experimental setup for Condition 1. **(Left)** Negative object demonstration by adult; **(Middle)** positive object demonstration by adult; **(Right)** infants’ interaction with objects. Written informed consent was obtained for the publication of this image.

In this condition, mothers were asked to teach their infants about the social value of pairs of ambiguous novel objects. For each pair of objects, mothers were instructed to describe one object with positive affect (“This is great, we really like this one!”) and the other object with negative affect (“This is bad, we don’t like this one”), as shown in **Figure 1**. Mothers were asked to limit their verbal descriptions to four simple formulaic sentences per object (which they repeated for each pair of objects), and to model positive or negative emotions in a prescribed manner (e.g., smiling versus frowning). The order of object presentation (positive or negative) was counterbalanced across trials. After observing their mothers’ teaching about both objects, infants were then allowed to interact briefly with the objects themselves before the objects were retrieved. During the session, an experimenter was present to ensure that participants were interacting as instructed. She provided new pairs of objects as required, but explicitly avoided making prolonged social contact with either participant.

#### Condition 2 (Play-Conducive)

**FIGURE 2:**
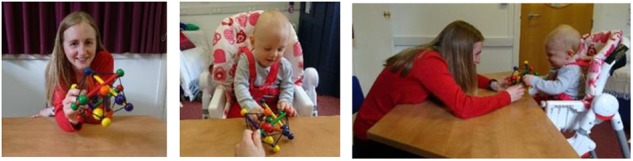
Illustration of experimental setup for Condition 2. **(Left)** Infants’ view; **(Middle)** adults’ view; **(Right)** side view of social interaction. Note that although the actors here are not wearing EEG caps, EEG signals were also collected during this condition. Written informed consent was obtained for the publication of this image.

In this condition, mothers were asked to play with their infant using a set of attractive toys (see **Figure 2**). Mothers were instructed to use the toy objects in a spontaneous, fun and natural way, to actively engage the infant’s attention, but to play quietly whilst avoiding large physical motions (in order to minimize EEG motion artifacts). During the session, an experimenter was present to ensure that participants were playing as instructed. She provided new toys as required (approximately every 2 min, or more frequently if the child threw the object to the floor) to sustain their attention and interest. The experimenter avoided making prolonged social contact with either participant.

### Video Recordings

To record the actions of the participants, two Logitech High Definition Professional Web-cameras (30 frames per second) were used, directed at the adult and infant respectively. Afterward, each video recording was manually coded for the timing of the behaviors of interest, using the coding scheme outlined in the Section “A Dimensional Framework for Analyzing Adult–Infant Play Patterns.” EEG data were also concurrently collected from mothers and infants during social interactions, but this data is not reported here as the primary focus of the current study is to develop a framework for assessing play behavior.

### A Dimensional Framework for Analyzing Adult–Infant Play Patterns

#### Coding Scheme

The intention of the coding scheme is to reliably capture common forms of playful behavior whilst retaining clarity of coding (grounding the scheme in clear, observable, behaviors). Accordingly, the minimization of false positives (non-play states coded as play-congruent) was prioritized over the minimization of false negatives (play states coded as play-incongruent). This was aided by our observations that false negatives in infants’ play tended to be rare and short in duration – e.g., throwing an object, directing focussed attention to something other than the play partner or play object. With this in mind, the coding scheme captures object-oriented play behavior in three dimensions: the socioemotional dimension (SE), the cognitive dimension (C), and the sensorimotor dimension (SM). On each dimension, simple, observable behavior at each timepoint (here, at the temporal resolution of 33 ms, corresponding to 30 frames per second) is coded using a [1/0] main code which indicates the presence or absence of play-congruent activity along the target dimension. Additionally, and where relevant, a further sub-code [1/0.x] may be assigned to indicate the level/type of activity that is occurring. These sub-codes (although not the focus of the current analysis) permit the capture and differentiation of more complex patterns of behavior in each dimension. The term ‘play-congruent’ is used to refer to behaviors and states in each dimension during which play might be occurring, and where the individual might be in a playful mental frame. In each dimension, the presence of play-congruent behavior is allocated a code of 1 and the absence of play-congruent behavior is allocated a code of 0. When play-congruent behavior is *concurrently* observed across all three dimensions (i.e., [1 1 1]), the resulting state is termed a ‘play-congruent state.’ The coding scheme is summarized in **Table 1**, and described further in the following text.

**Table 1:** Coding scheme.

Dimension	Behavior	Code	Sub-code
Socioemotional (SE)	Neutral affect	1	1
	Display of positive affect e.g. wide-eyed, smiling/laughing, happy vocalizations, energetic movements, raised eyebrows.		2
	*Display of negative affect e.g. frowning, uninterested, sad, fussing, crying*	0	1
	*Affect unclear (e.g., baby obscured/looks away/no sound) and affect is different when looks back or sound returns*		2
Sensorimotor (SM)	Physical contact with object and no actions (e.g., holding)	1	1
	Physical contact with object and circular/repetitive action/motion with object (e.g., banging, shaking, mouthing)		2
	Physical contact with object and other (non-circular/non-repetitive) action with object		3
	*No contact with object or passive contact*	0	-
Cognitive (C)	Attention is focused on the object or partner with no additional intentional behavior related to object	1	1
	Attention is focused on the object or partner with non-object-specific exploration of object (banging, shaking, mouthing)		2
	Attention is focused on the object or partner with object-specific exploration of object		3
	Attention is focused on the object or partner with pretense/acting behavior related to object		4
	Attention is focused on the object or partner with rule-based behavior related to object		5
	*Attention is not on the object or partner*	0	-

*Please see text for a detailed description and explanation.*

##### Socioemotional (SE)

The presence of positive affect and the idea that play is done for its own sake, leading to an internal sense of reward rather than any form of external reward, are both central to most conceptualizations of play behavior (Burghardt, 2005; Miller, 2017). However, whilst negative affect is considered antithetical to the presence of a mental ‘play-state,’ a neutral display of affect could also be present during play (Miller, 2017). Therefore, the expression of positive or neutral affect was taken as congruent with a play-state in the socioemotional dimension.

##### Sensorimotor (SM)

The sensorimotor dimension captures whether or not there is voluntary physical contact with the object that is free from external constraint. It is not possible to engage in object play without physical contact with the object, so this dimension encodes a necessary condition for one of the main play behaviors of interest during infancy. Furthermore, the fact that only voluntary contact is coded reflects the central place of self-directed, voluntary behavior in definitions of play (Burghardt, 2005; Lillard et al., 2013; Miller, 2017; Sawyer, 2017). Therefore, voluntary physical contact with the object was deemed as congruent with a play-state in the sensorimotor dimension. The primary limitation of this criterion is that it will not capture play behavior with no physical contact with the object – for example, when throwing or dropping objects. However, in infancy, play behavior without physical contact is rare: even if an infant drops or throws a toy, they tend to either pick it up again soon after, or cease playing with it. It is only later in life that forms of play appear which can involve sustained lack of contact, e.g., sociodramatic play and games with rules. Consequently, confining positive coding in the SM dimension to physical contact may be conservative but only rarely erroneous.

##### Cognitive (C)

The cognitive dimension captures the level of cognitive complexity and engagement, by coding whether or not there is visual attention on the object and/or play partner and what kind of behavior is occurring in relation to the object. While playful behavior may occur without active attention on the object or play partner (for example, an infant swinging a toy around while not looking at anything in particular, or with eyes closed), we decided that, as we were interested in play’s effects at the neural level, even our broadest criteria for a play-congruent state should include some level of cognitive engagement. In other words, our model is intended to explore ‘minds-on’ play, rather than ‘minds-off’ play that is purely physical or sensory in nature. During infancy, looking behavior is closely related to visual attention, and is frequently used as an index of early emerging cognitive function and development (Colombo, 2001). Therefore, visual attention on either the object or the play partner (as determined by participants’ looking behavior) was deemed as congruent with a play-state in the cognitive dimension.

**FIGURE 3:**
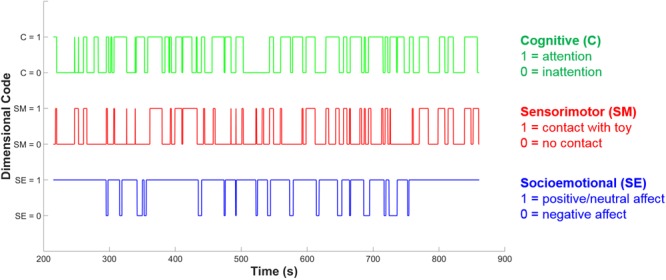
Example of dimensional coding over time. The blue line shows socioemotional (SE) dimension coding, the red line shows sensorimotor (SM) dimension coding and the green line shows cognitive (C) dimension coding. At each time point, each dimension may either be coded as 0 or 1, indicating the absence or presence of play-congruent dimensional activity.

In the cognitive dimension five sub-codes were also developed to delineate whether the individual is also engaged in exploratory behavior (object-general or object-specific), pretense or acting, or rule-based behavior. The distinction between object-general and object-specific exploration is intended to capture two different levels of cognitive engagement which may relate to observable differences in neural activity. Object-general exploration is any kind of activity with the object that does not involve appreciation of the object’s particular properties, i.e., the action could be done with almost any object. The main examples of object-general exploration include shaking, banging, or mouthing the object, and these behaviors are often done in a repetitive or circular fashion. Object-general exploration may provide sensory stimulation and coarse, ‘global’ information, such as object weight or texture, but seems unlikely to lead to specific conceptual information about an object’s functions and uses. Object-specific exploration, by contrast, involves an appreciation of that object’s unique properties – for example, spinning the blades of a toy helicopter, or pulling on parts of the object to see if they can be removed. It is this kind of exploration that seems most likely to involve the processing of more complex information, and lead to more advanced conceptual learning about an object’s functions and uses. This distinction between object-general and object-specific behavior is parallel to the distinction made by Belsky and Most (1981), between mouthing or simple manipulation and ‘functional play’ which is appropriate for the specific object. Belsky and Most (1981) found object-specific play to be more developmentally advanced than more general, non-specific object play, supporting our decision to encode object-specific play as a more cognitively engaged form of play in our coding scheme. **Figure 3** shows an example of the resulting codes for each separate dimension over time during a social interaction episode for an infant.

#### Coders and Reliability

Coding of all videos was performed by one trained coder. To establish inter-rater reliability, approximately 20% (48 min) of the infant and adult video data was coded by a second coder, who was trained independently from the first coder. Percentage agreement was over 90% on all 3 dimensions for the infant (*SE* = 91%, *SM* = 98%, *C* = 95%) as well as for the adult (*SE* = 97%, *SM* = 94%, *C* = 93%), indicating a high level of agreement.

### Analysis of Social States

#### Mean Dimensional Scores

For each dimension (socioemotional [SE], sensorimotor [SM], cognitive [C]), separate SE, SM and C mean dimensional scores can be computed by taking the average over all timepoints in the session. This mean score ranged between 0 (if all timepoints were coded as 0) and 1 (if all timepoints were coded as 1). Accordingly, if infants generally displayed more positive/neutral affect than negative affect, their SE mean score would be greater than 0.5 (i.e., higher proportion of 1 s than 0 s overall). Similarly, the mean SM score indicates the proportion of time during which the infant has active “hands-on” possession of the toy (e.g., mean SM score of 0.7 = infant has active possession of the toy 70% of the time). Finally, the mean C score indicates the relative attentiveness of the infant during the session (e.g., mean C score of 0.6 = infant is attentive toward the object or partner 60% of the time). It is important to emphasize that a high score on a single dimension (or indeed across several dimensions) does not in itself indicate that the infant is highly engaged in play, since these mean dimensional scores are independent of each other and therefore provide no information about *temporal co-occurrence* across dimensions (i.e., social state). For example, it would, in theory be possible for the infant to show positive affect *only when* inattentive/not touching the toy and yet still produce a high mean score on the SE dimension.

#### Social States (Per Timepoint)

**FIGURE 4:**
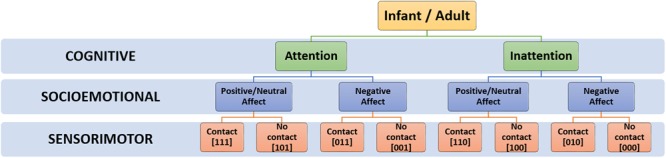
Hierarchical taxonomy of possible social states.

At each timepoint, an infant’s current social state can be defined by the temporal co-occurrence and valence of existing codes on each dimension, signifying affect (SE), touch (SM) and attention (C) respectively. As SE, SM and C codes could each take a value of 0 or 1, this allows a total of 8 distinct social states (i.e., 2^3^), as outlined in **Table 2** and **Figure 4**.

**Table 2:** Examples of possible social states and their potential interpretation.

Social state	Dimension main code			Interpretation
	*SE*	*SM*	*C*	
1	1	1	1	Positive/neutral affect, contact and attention ***(=Play-congruent state)***
2	1	0	1	Positive/neutral affect, no contact, attention
3	0	1	1	Negative affect, contact, attention
4	0	0	1	Negative affect, no contact, attention
5	1	1	0	Positive/neutral affect, contact, inattention
6	1	0	0	Positive/neutral affect, no contact, inattention
7	0	1	0	Negative affect, contact, inattention
8	0	0	0	Negative affect, no contact, inattention

**FIGURE 5:**
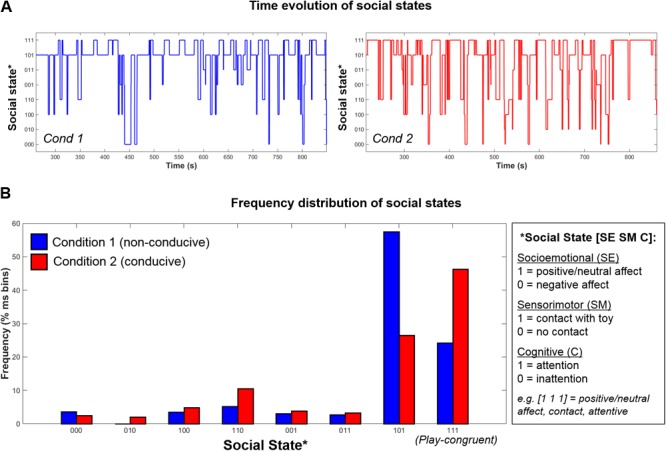
Example data from one infant showing the **(A)** Time-evolution between 8 possible social states during Condition 1 (left, blue) and Condition 2 (right, red); **(B)** Frequency distribution of social states in Condition 1 (blue) and Condition 2 (red).

Further, as social states were computed for every time-point in the session, this permits the tracking of infants’ dynamic evolution between social states over time, as illustrated in **Figure 5A**, as well as the relative proportion of time that infants spend in each state (**Figure 5B**). Finally, using the state frequency histogram, it is possible to identify the *modal* social state for a given interaction session.

#### Adult–Infant Joint States (Behavioral State Synchrony)

The *joint* (i.e., concurrent) social state of adults and infants can also be assessed using this scheme. For example, during didactic teaching, only the cognitive dimension may be concurrently engaged in both partners, whilst their sensorimotor and socio-emotional states may be discordant (e.g., Mother’s state is [1 0 1] whilst the infant’s state is [0 1 1]). This may also be performed to examine joint states within a particular dimension (e.g., affect), considered alone. Such joint state analysis could be useful to address research questions pertaining to parent-child synchrony (since if both parent and child display the same state at the same time, they are behaving synchronously), contingency and responsiveness.

Finally, this framework permits an empirical discrimination between similar/related social interactional states such as teaching versus play. Although the play-congruent state [1 1 1] is of greatest interest here, a total of 8 different individual states for infants and adults (and 64 joint adult-infant states) may be discriminated under the proposed framework, which may yield insights into a new biologically grounded taxonomy of play interactions.

## Results

Here, we report the results from the *main* codes assigned along each dimension (e.g., 1 or 0 – see **Table 1**). However, if desired, more fine-grained information about the quality of social interaction may be gleaned by examining participants’ sub-codes, as detailed in the Supplementary Materials.

### Mean Dimensional Scores

**FIGURE 6:**
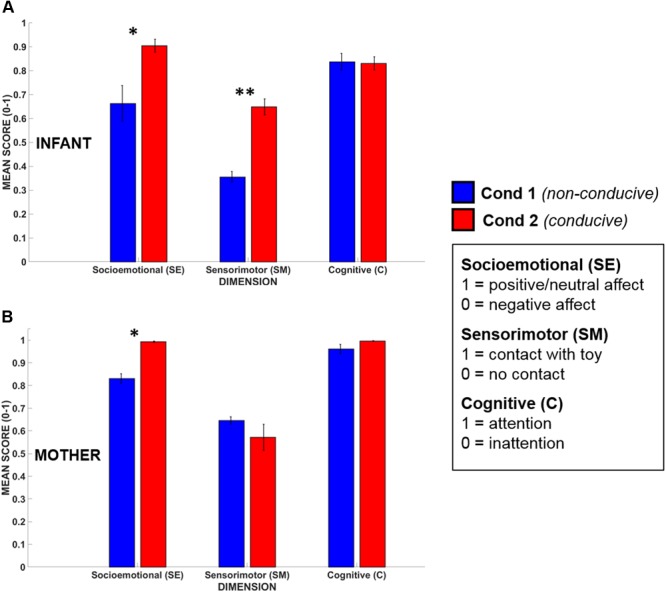
Mean dimensional scores for **(A)** infants’ and **(B)** mothers’ in Condition 1 (blue) and Condition 2 (red). Error bars indicate the standard error of the mean. ^∗^*p* < 0.05, ^∗∗^*p* < 0.01.

Infants’ (*N* = 5) and mothers’ (*N* = 5) mean dimensional scores obtained during Conditions 1 and 2 are shown in **Figure 6**. All scores were normally distributed (Kolmogorov–Smirnov test, *p* > 0.20 for all dimensions). The data were assessed statistically using a Repeated Measures ANOVA taking Dimension (3 levels, SE/SM/C) and Condition (2 levels) as within-subjects factors, and participant (infant or mother) as a between-subjects factor.

The ANOVA revealed that there was a significant main effect of Condition [*F*(1,8) = 18.82, *p* < 0.01, $\eta_{p}^{2}$ = 0.70), where, across all dimensions, mean dimensional scores for Condition 2 (play-conducive) exceeded those for Condition 1 (not play-conducive). Importantly, there was also a significant interaction between Condition and Dimension, suggesting that not every dimension differed between Conditions [*F*(2,16) = 8.42, *p* < 0.01, $\eta_{p}^{2}$ = 0.51]. Tukey HSD *post hoc* analysis of this interaction indicated that Socioemotional and Sensorimotor dimensional scores were both significantly higher during Condition 2 than Condition 1 (*p* < 0.001, *p* < 0.05 respectively), but Cognitive dimensional scores did not differ (*p* = 0.99). Therefore, during social interactions that supported playful behavior, both infants and their mothers (on average) showed more positive/neutral affect and active possession of the toy, but they were equally attentive across both conditions. There was also a significant main effect of Participant [*F*(1,8) = 54.7, *p* < 0.001, $\eta_{p}^{2}$ = 0.87] with mothers showing higher dimensional scores overall than their infants, which is consistent with high compliance and task-engagement by adults.

Whilst dimensional scores are able to capture time-averaged differences in overall social interactional quality, they cannot reveal whether qualitatively-different *types* of social interaction are occurring (as well as their timing and frequency of occurrence). Accordingly, we next assessed infants’ social states (calculated for each timepoint) in each condition.

### Frequency Distribution of Social States During Play and Teaching

**FIGURE 7:**
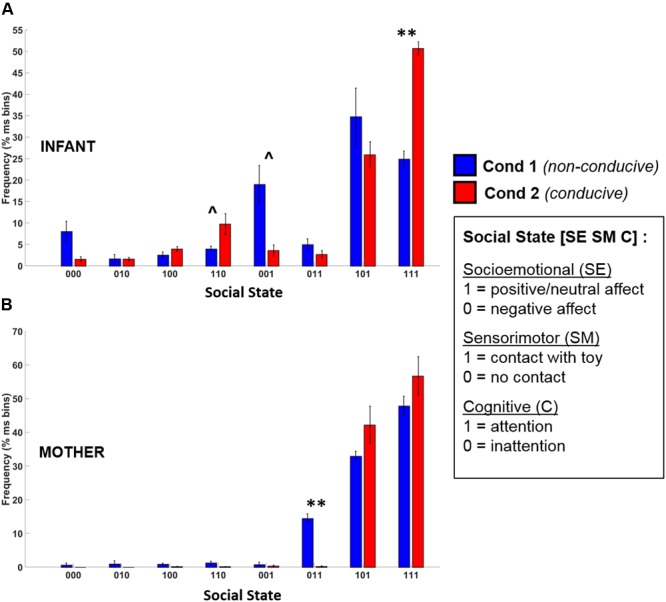
Frequency distribution of the eight possible social states for **(A)** infants and **(B)** mothers across Condition 1 (blue) and Condition 2 (red). Error bars indicate the standard error of the mean. ^∗∗^*p* < 0.01, ˆ*p* = 0.12 (BH-FDR corrected).

The frequency distribution of different social states observed in infants and mothers during Conditions 1 and 2 are shown in **Figure 7**. Given that there were only 5 data points, this provided insufficient degrees of freedom to conduct an omnibus Repeated Measures ANOVA. Accordingly, to assess whether there were statistical differences between conditions in social state frequency, we conducted paired *t*-tests (Benjamini–Hochberg FDR corrected *p*-values, α = 0.05) for each social state, for infants and mothers.

#### Infants

The *t*-test results revealed that there was a large and significant increase in the frequency of the play-congruent [1 1 1] social state during Condition 2 as compared to Condition 1 [*t*(4) = 8.97, BH-FDR *p* < 0.01, *d* = 4.01]. On average, during Condition 1, infants were in a [1 1 1] state 24.9% of the time but during Condition 2, this frequency doubled to 50.7% (i.e., half) of the total time spent in social interaction. Similarly, during Condition 2, there was a trend toward an increase in the frequency of the [1 1 0] social state [*t*(4) = 2.90, BH-FDR *p* = 0.12, *d* = 1.30], and a similar trend toward a decrease in the frequency of the [0 0 1] social state [*t*(4) = -3.11, BH-FDR *p* = 0.12, *d* = 1.39]. Together, these results suggest that during Condition 2 (which was conducive to playful behavior), infants spent significantly more time in social states characterized by “positive-affect hands-on interaction” (i.e., [1 1 1] or [1 1 0]), and proportionately less time in a negative-affect passive observational state [0 0 1].

#### Mothers

Mothers’ *t*-test results revealed only one significant difference between conditions. There was a significant decrease in the frequency of the play-incongruent social state of [0 1 1] (negative affect, contact, attention) in Condition 2 as compared to Condition 1 [*t*(4) = -9.85, BH-FDR *p* < 0.01, *d* = 4.41]. However, although there was a trend toward an increase in play-congruent behavior ([1 1 1]) in mothers for Condition 2, this increase was not significant [*t*(4) = 1.28, BH-FDR *p* = 0.43, *d* = 0.57]. Therefore, although mothers displayed less play-*in*congruent behavior during Condition 2 than Condition 1, we did not observe significantly more play-congruent behavior.

### Individual Modal States

#### Infants

During Condition 1, the modal (most frequently occurring) state was [1 0 1] for 4 infants, and [0 0 1] for 1 infant. However, during Condition 2, the modal state for all 5 infants was the play-congruent state of [1 1 1]. Therefore, there was a clear difference in the characteristic state of infants between conditions. During social interactions that were non-conducive to playful behavior, infants were predominantly passive (“hands-off”) but attentive. During social interactions that supported playful behavior, infants were predominantly active (“hands-on”), positive and attentive.

#### Mothers

By contrast, mothers displayed almost no difference in their modal states across experimental conditions. Four out of five mothers showed a modal state of [1 1 1] for both Conditions 1 and 2. One mother showed a modal state of [1 1 1] during Condition 1, and a modal state of [1 0 1] during Condition 2, with [1 1 1] being her next most frequently occurring social state.

### Mother–Infant Joint States (Behavioral State Synchrony)

**FIGURE 8:**
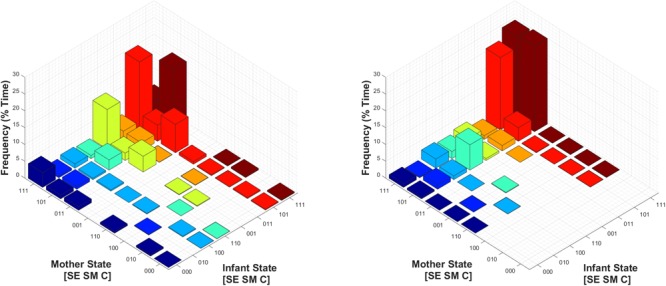
Mean joint probability distribution of mothers’ and infants’ social states during Condition 1 **(Left)** and Condition 2 **(Right)**. The joint play-congruent state of [1 1 1] – [1 1 1] for mother and infant respectively is shown in the posterior corner of each subplot, and shaded in dark red.

Finally, we assessed the joint probability distribution of infants’ and mothers’ social states during Conditions 1 and 2, as shown in **Figure 8**. Of note, perhaps the most relevant difference is that during Condition 1, mothers and infants were in a joint play-congruent social state (i.e., [1 1 1] – [1 1 1], or *synchronous play*) only 5.7% of the time on average. By contrast, during Condition 2, mothers and infants showed synchronous play 24.9% of the time – a nearly fivefold increase. Therefore, during conducive social contexts (Condition 2), the play-congruent state occurred more frequently in regard to infants’ own behavior, and this joint social state also occurred concurrently (i.e., synchronously) with their mothers more often.

## Discussion and Conclusion

Play behavior is diverse and multi-faceted, and a major challenge for current research is to converge on a common definition and measurement system for play that integrates behavioral, cognitive and neurological levels of analyses. Here we present and test a new methodological framework that captures different social interactional states (play-congruent or play-incongruent) and permits an empirical discrimination between similar and related social interactional states (such as joint activities in situations both conducive and non-conducive to the emergence of playful behavior).

*A priori*, we made two sets of predictions about the differences in infants’ behavior between these conditions. Our coding results supported both predictions. First, we observed that, during conducive social interactions designed to be conducive to play (Condition 2), infants’ modal state was indeed coded as play-congruent (i.e., [1 1 1]). Further, infants spent significantly more time in social states characterized by “positive-affect hands-on interaction,” and proportionately less time in a negative-affect passive observational state. This result demonstrates the *sensitivity* of the coding scheme in correctly identifying the intended mode of social interaction as either playful or non-playful, even though coders did not explicitly code for play itself. Further, our data also highlight the fact that, although mothers were instructed to play with their infants during Condition 2, infants themselves did not display playful behavior *all of the time* (on average, only 50.7% of the time). Rather, infants showed a heterogenous mixture of social states characterized variously by positive affect and “hands-on” engagement. This behavioral finding has important practical implications for the analysis of infants’ *neural* data that is collected during such play sessions. If infants’ neural data during play is assumed to be homogenous and analyzed as such (e.g., by computing averages of neural indices across all time-points), such an analysis would be erroneous as infants may be engaging in different forms of play (as well as other related types of social interactions) over a period of time. The proposed coding framework therefore lends itself well to time-sensitive neural analyses, because it permits the automatic extraction of discrete time periods when a certain social state is observed (i.e., [1 1 1]), as well as separate analyses of the periods leading up to, and away from these moments.

As predicted, our coding results also showed that, when infants were engaged in social interactions that were designed to be conducive for playful behavior (Condition 2), they showed decreased negative affect, increased sensorimotor involvement with objects, but equivalent attentional engagement. Similarly, mothers also showed significantly decreased negative affect during social interactions that supported playful behavior. Mothers’ decrease in negative affect during Condition 2 was expected as they had been instructed to model both positive and negative affect in Condition 1. However, it is interesting to note that their infants also showed a similar decrease in negative affect. This result also demonstrates the potential utility of the coding scheme in highlighting key differences between different forms of early social interactions. Specifically, the finding that infants were no less cognitively engaged during interactions where the mother was not explicitly teaching her infant suggests that playful interactions might provide an equally (if not more) effective social context for early learning as compared to direct didactic instruction from parents.

Finally, we observed that during play, parent-child dyads showed greater temporal synchrony with each other’s social states, as mothers and infants were concurrently (jointly) in a playful state (i.e., [1 1 1] – [1 1 1]) five times more frequently than was observed during teaching. This strong alignment of social-affective state between parent and child during playful scenarios is consistent with previous work. For example, patterns of temporally synchronous activity between parent and child during social interaction have been noted for gaze (Kaye and Fogel, 1980), affect (Cohn and Tronick, 1988; Feldman et al., 2011) and even autonomic arousal (Feldman et al., 2011; Waters et al., 2014). Our coding scheme not only allows the identification of specific time periods when play is synchronously occurring between mother and child (e.g., for neural analyses), but also allows comparison to periods when the dyad is socially asynchronous, or ‘out of tune’ with each other. Such parent-child asynchrony is known to occur more frequently and to be of particular clinical relevance in affective disorders such as maternal depression (Goldsmith and Rogoff, 1997; Jameson et al., 1997) which is known to have an impact on the quantity and quality of children’s own play (Murray et al., 1999). However, two caveats should be noted when interpreting these synchrony data. First, ‘social state synchrony’ (as defined by our coding scheme) may not necessarily imply that mother and infant are jointly engaged in the same activity. The current scenario involved play with a single toy object, however, if multiple toy objects were present, parent and child could be interacting separately with different objects yet still be coded as being in a joint state of play. Second, it should be noted that successful social interactions also include more complex temporal contingencies (e.g., turn-taking) where partners’ actions are not concurrent (de Barbaro et al., 2013b; Leclère et al., 2014). As the current coding scheme captures the temporal evolution of different states, in future, these non-synchronous temporal contingencies between parents and children could also be identified and examined.

### Limitations

One major limitation to the current study is its small sample size and the restricted movement of participants (which was necessary for concurrent EEG measurements but could reduce ecological validity). However, our intention has been to illustrate the sensitivity of the proposed framework in discriminating between different play-related states, using a coding scheme grounded in simple, observable behavior and with a temporal resolution suited to neuroscientific research. Five dyads provided sufficient data to assess the efficacy of the framework as a means of capturing variations in individual and joint behavior as continuously evolving states, rather than as discrete actions or a subjective global assessment. Nevertheless, research applying our framework to larger samples is needed to ensure that the contextual differences we identified between play and teaching scenarios are generalisable.

A second limitation is our focus on one specific type of play which revolves around a physical object. However, the focus on a physical object does not limit our model entirely to earlier and more basic types of play, because a physical object can be used in play with more symbolic content. For example, object substitution – using an object as if it is something else – is often coded in established play coding schemes as an indicator of pretend play. Many games with rules involve physical objects, so participants could engage in such a game, either spontaneously or because they are asked to do so. We decided to capture these more complex types of behavior in our model, with the acknowledgment that in infancy, these types of behavior will be very rare, and most likely observed on the part of the parent. Also, with appropriate development (e.g., the elaboration of sub-code options) our framework could be applied to more abstract forms of play that do not revolve around physical objects. It may also be possible to analyze play with multiple objects, although this would make the coding of dyadic states more complex, because it could no longer be assumed that the dyad were playing together if they showed the same state (for example, parent and child may each be touching and engaging with a different toy yet both show the [1 1 1] state). However, the scheme can be used in its present form to code solo play. Comparing behavioral and neurological results from solo play with multiple objects to solo play with one object could provide important developmental insights, as there is evidence that play with multiple objects is developmentally distinct from play with a single object and arises later in ontogeny (Belsky and Most, 1981).

A third potential limitation is the use of looking behavior as the primary index of cognitive engagement. Infants may stare at something without much cognitive engagement, and several researchers have discussed the limitations of using looking behavior to index cognitive engagement during infancy (Ruff and Rothbart, 1996; Aslin, 2007; Richards, 2010). Richards has, for example, distinguished between looks that are accompanied by concomitant physiological changes from those that are not, and found that the degree of physiological change is a better indicator than the mere presence of looking behavior as to whether information presented is retained (e.g., Richards and Casey, 1991). Ruff has differentiated different qualities of visual attention based on detailed video coding (Ruff and Capozzoli, 2003). Nonetheless, during object play, looking behavior can be a useful indicator of infants’ level of cognitive engagement. For example, in an analysis of infant exploratory behavior with objects, Caruso (1993) found that ‘sophisticated exploration,’ which included visual examination and manipulating an object to look at it, was negatively related to mouthing and gross motor manipulation with objects (termed ‘unsophisticated exploration’). Thus, in periods where the infant exhibits sustained attention on the parent and/or toy object, it seems likely that they are engaging in social interaction and/or attempting to understand or manipulate the toy object. Similarly, a child could have an object in their mouth and therefore not be looking at it, but this is what we regard as a more sensory, ‘minds-off’ form of play, where the action (mouthing) and visual attention are directed to different stimuli, i.e., there is a *divided* sensory focus compared to an infant whose action and attention are congruent, through both looking at a toy and reaching for or holding a toy. Therefore, despite limitations, for practical reasons, and in common with numerous other researchers in the field (e.g., Yu and Smith, 2016), we have taken the simple presence or absence of looking behavior toward an object as an indicator of cognitive engagement. However, it should be noted that for adults, this might be an unnecessarily restrictive definition of cognitive activity.

A final limitation is that, as play behavior changes significantly across the life-span (Power, 1999), we chose to focus primarily on infants. Therefore, adult play behavior may not have been optimally captured by the framework. Nonetheless, our coding revealed an interesting result: mothers (unlike their infants) did not show a clear shift toward greater playfulness for Condition 2 as compared to Condition 1, although their negative affect decreased overall. One possible reason for this may be that mothers approached the teaching exercise in Condition 1 as pretend play. Since the objects used in Condition 1 had no intrinsic social value, mothers had to act out a ‘good’ or ‘bad’ response to the objects, by pretending that the objects had a particular social significance. As this was not a classic pretend play situation (in that infants might not know that their mothers were pretending), this could explain why infants (aged on average 10.7 months) responded less playfully to their mother’s social pretend play. The result would also be consistent with the late emergence of pretend play capabilities during the second year of life (Fein, 1981).

### Toward a Neuroscientific Understanding of Play

Although the neural EEG data that was collected during the parent–child social interactions was not analyzed here, the proposed framework represents an important first step toward analyses of the concomitant neural data, which is planned for future investigation. Specifically, since the proposed framework fractionates play behavior along dimensions that have previously been associated (in animal studies) with well-defined neural circuits, this provides the potential to generate specific hypotheses regarding the neural activation patterns predicted to accompany each of the 8 possible social states. An analysis of these underlying neural substrates may, in future, lead to a definition of play behavior that is more closely grounded in neuroscience.

**FIGURE 9:**
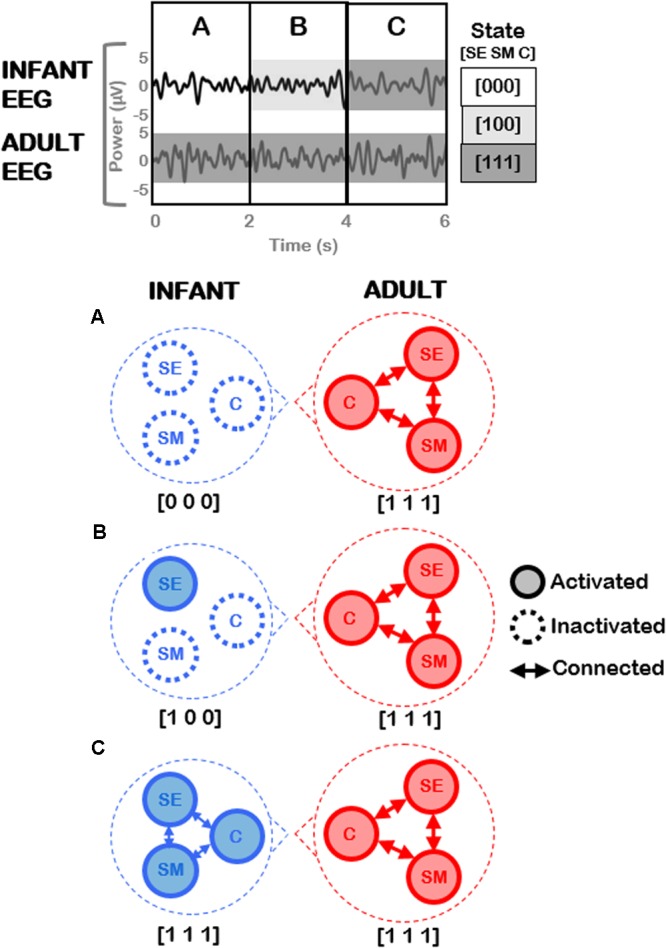
Illustration of future planned EEG analysis utilizing current coding framework. **(Top)** Hypothetical example of EEG signals concurrently measured from an infant and an adult during joint play. Three different time periods (A, B, C) may be identified based on different social states in the infant, assuming that the adult remains in a constant play-congruent state [1 1 1]. **(Bottom)** Highly-simplified and illustrative predictions of the patterns of neural activation expected during each time period, for infant **(Left)** and adult **(Right)** respectively. Each node represents a neural circuit subserving each dimension (socioemotional [SE], sensorimotor [SM], and cognitive [C]). Arrows represent functional connectivity between these respective neural circuits.

**Figure 9** provides an illustration of potential analyses that could be conducted on the adult-infant EEG data, and (highly simplified) examples of neural activation patterns that could underpin three different social states (including the play-congruent [1 1 1] state). The top panel of **Figure 9** depicts EEG signals concurrently measured from infant and adult during the joint play session. After the application of behavioral coding to the accompanying video, the EEG data may be divided into three different time periods (A, B, C) based on the infant’s social state (for simplicity, here the adult remains in the play-congruent state [1 1 1] throughout). The bottom panels show the predicted patterns of neural activation that would be expected during each time period, for the infant and the adult. For simplicity (and purely illustrative purposes), here, each circled node represents a neural circuit that subserves each dimension (socioemotional [SE], sensorimotor [SM], and cognitive [C]). It may be hypothesized that each neural circuit will show activation [1] or inactivation [0] depending on the concomitant behavioral state of the participant. Further, when more than one neural circuit is activated, these circuits may show mutual patterns of functional connectivity, as represented by connecting arrows in the figure. In this case, the play-congruent [1 1 1] state would also be associated with the highest levels of neural activation and connectivity across brain regions. Note that in this example, changes in the infant’s behavioral state (e.g., from [1 0 0] to [1 1 1]) occur only every 2 s. However, the coding scheme permits precise identification of the start and end points of each social state with a temporal precision of 10s of milliseconds (i.e., 2.1 s versus 2.4 s) which is crucial for phase-based connectivity analyses of EEG oscillatory signals.

### Conclusion

In conclusion, we have presented a novel dimensional framework for analyzing the continuous time-evolution of adult-infant play patterns, underpinned by biologically informed state coding along sensorimotor, cognitive and socio-emotional dimensions. We expect that the proposed framework will have wide utility amongst researchers wishing to employ an integrated, multimodal approach to the study of play, and lead toward a greater understanding of the neuroscientific basis of play.

## Ethics Statement

This study was carried out in accordance with the recommendations of Cambridge Psychology Research Ethics Committee with written informed consent from all subjects. Parents gave written informed consent on behalf of their children in accordance with the Declaration of Helsinki. The protocol was approved by the Cambridge Psychology Research Ethics Committee.

## Author Contributions

DN designed the coding scheme and wrote the article. KC and SG collected the data. HD analyzed the data. MS helped to design the coding scheme and wrote the article. SW designed the experiment and wrote the article. VL designed the experiment, analyzed the data, and wrote the article.

## Conflict of Interest Statement

The authors declare that the research was conducted in the absence of any commercial or financial relationships that could be construed as a potential conflict of interest.
