# Supplementary material for: Toward a Neuroscientific Understanding of Play: A Dimensional Coding Framework for Analyzing Infant–Adult Play Patterns
Source: Front Psychol. 2018 Mar 21;9:273. doi: 10.3389/fpsyg.2018.00273 (PMC5871690; doi:10.3389/fpsyg.2018.00273)
Supplement: Supplementary file 1 [file Data_Sheet_1.DOCX]

**SUPPLEMENTARY MATERIALS**

The assignment of sub-codes allows for even more precise discrimination of the quality of social interaction. For example, consider periods of activity coded in the 3 behavioural dimensions in the following ways:

**Examples of play-congruent-states:**

**a) Joint attention on the object/partner, holding object, positive affect:**


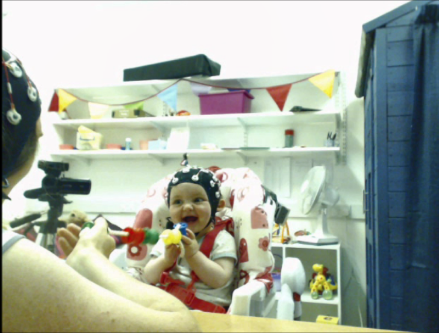

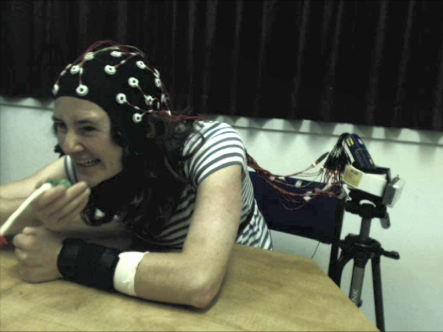


**SE SM C**

**Parent 1.2 1.1 1.1**

**Child 1.2 1.1 1.1**

**b) Joint attention on the object/partner, holding object, neutral affect:**


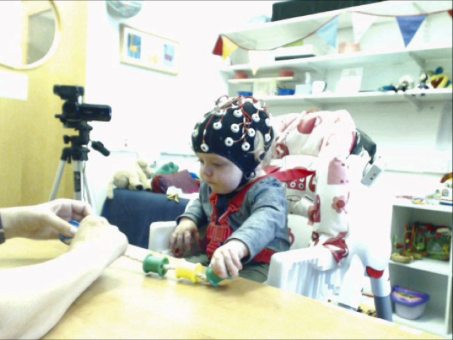

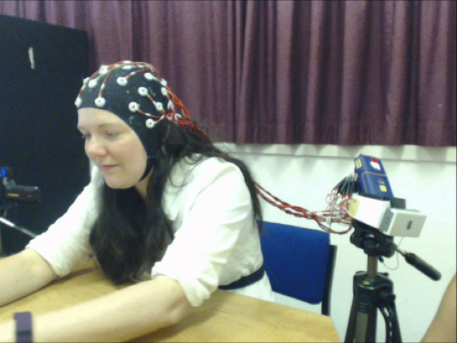


**SE SM C**

**Parent 1.1 1.1 1.1**

**Child 1.1 1.1 1.1**

**c) Solo object-specific exploration by child, neutral affect**


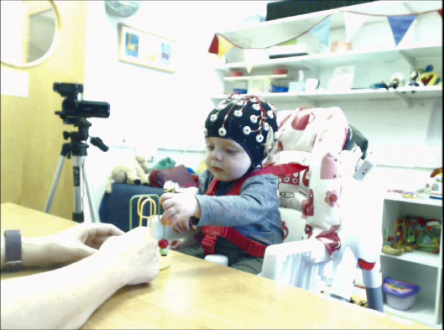

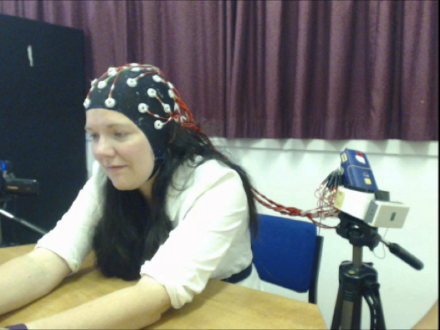


**SE SM C**

**Parent 1.1 1.1 1.1**

**Child 1.1 1.3 1.2**

**d) Solo object-specific exploration by child, positive affect**


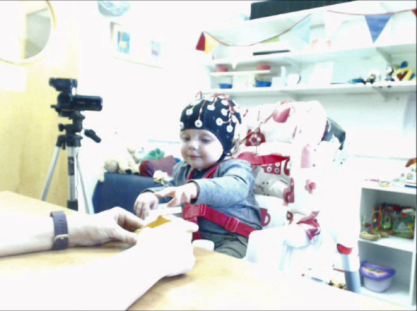

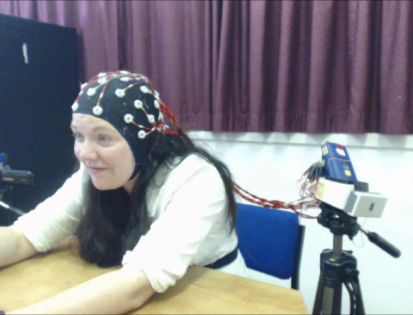


**SE SM C**

**Parent 1.2 1.1 1.1**

**Child 1.2 1.3 1.2**

**e) Joint object-specific exploration plus display of positive affect:**


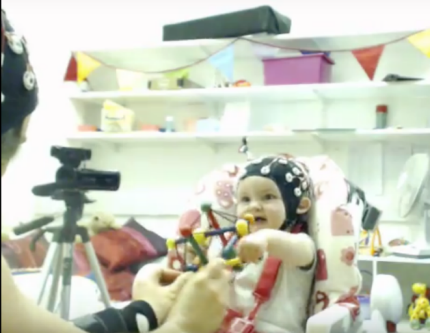

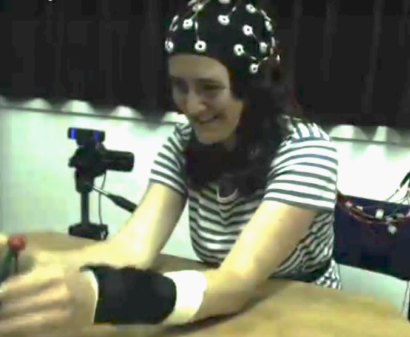


**SE SM C**

**Parent 1.2 1.3 1.2**

**Child 1.2 1.3 1.2**

**Example of a play-incongruent state:**

**f) Both holding toy, positive affect, child distracted:**


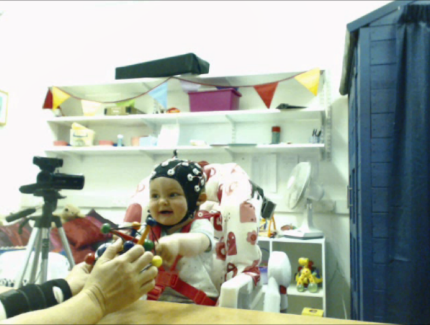

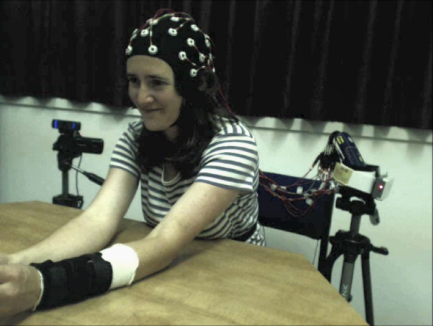


**SE SM C**

**Parent 1.2 1.1 1.1**

**Child 1.2 1.1 0**
